# Prevalence of positive depression screen among post miscarriage women- A cross sectional study

**DOI:** 10.1186/s12888-018-1619-9

**Published:** 2018-02-05

**Authors:** Steve Kyende Mutiso, Alfred Murage, Abraham Mwaniki Mukaindo

**Affiliations:** Department of Obstetrics and Gynaecology, Aga Khan University hospital, Nairobi, Kenya

**Keywords:** post-miscarriage, depression, prevalence

## Abstract

**Background:**

Miscarriages are a common pregnancy complication affecting about 10–15% of pregnancies. Miscarriages may be associated with a myriad of psychiatric morbidity at various timelines after the event. Depression has been shown to affect about 10–20% of all women following a miscarriage. However, no data exists in the local setting informing on the prevalence of post-miscarriage depression. We set out to determine the prevalence of positive depression screen among women who have experienced a miscarriage at the Aga Khan University hospital, Nairobi.

**Methods:**

The study was cross-sectional in design. Patients who had a miscarriage were recruited at the post-miscarriage clinic review at the gynecology clinics at Aga Khan University Hospital, Nairobi. The Edinburgh postpartum depression scale was used to screen for depression in the patients. Prevalence was calculated from the percentage of patients achieving the cut –off score of 13 over the total number of patients.

**Results:**

A total of 182 patients were recruited for the study. The prevalence of positive depression screen was 34.1% since 62 of the 182 patients had a positive depression screen. Moreover, of the patients who had a positive depression screen, 21(33.1%) had thoughts of self-harm.

**Conclusion:**

A positive depression screen is present in 34.1% of women in our population two weeks after a miscarriage. Thoughts of self-harm are present in about a third of these women (33.1%) hence pointing out the importance of screening these women using the EPDS after a miscarriage.

**Electronic supplementary material:**

The online version of this article (10.1186/s12888-018-1619-9) contains supplementary material, which is available to authorized users.

## Background

A miscarriage is the premature loss of a fetus prior to viability. This may have different definitions depending on a regions’ definition of the cusp of viability but the World Health Organization (WHO) defines it as the premature loss of a fetus up to 23 weeks of pregnancy and weighing up to 500 g [1]. Miscarriages are among the most common complication of pregnancy occurring in about 10–15% of pregnancies that are considered low-risk [2, 3]. Most of these miscarriages occur in the first trimester with a steady decline on the frequency of miscarriages up to about 20 weeks [4]. More so, more than 75% of miscarriages occur prior to 18 weeks [4]. A miscarriage can either be spontaneous or induced in etiology [5]. Spontaneous miscarriages have a myriad of causes including chromosomal abnormalities, uterine anomalies and environmental agents such as alcohol intake or cigarette smoking [6, 7].

Whether they are spontaneous or induced miscarriages have a significant implication in psychiatric morbidity of the involved women [8, 9]. However, induced miscarriages have been associated with higher rates of psychiatric complications compared to spontaneous ones [10]. Psychiatric illnesses have been shown to be a major complication of miscarriages with various psychiatric morbidities being linked to it including depression, anxiety and even post-traumatic stress disorders [9, 11, 12]. Furthermore, such psychiatric illnesses have been shown to have an implication in subsequent pregnancies with women who have had psychiatric morbidity being shown to be at higher risk of redeveloping the conditions in pregnancy and also other psychiatric conditions [12].

Depression in women who have had a miscarriage occurs at various timelines after the miscarriage episode [13]. Starting as early as within 10 days and being able to last a woman’s lifetime, depressive illness seems to be of significant burden after a miscarriage [14]. Moreover, women experiencing a miscarriage have varied socio-cultural backgrounds as opposed to women going through a term pregnancy with a live outcome [15]. Furthermore, pregnant women in the region have also been shown to be of different socio-cultural backgrounds as compared to a Caucasian population [16]. Women in the region are married at an earlier age, with most living in the confines of an extended family and have wrong conceptions about the causation and implications of a miscarriage [4]. These varied factors argue out the importance of a regional study to look at the implications of miscarriages on depression. Various studies, conducted worldwide but none in Africa, have analyzed the prevalence of depression following a miscarriage with rates of 10–20% being described [14, 17]. Depression has also been shown to be significantly higher after a miscarriage compared to women who have not had a history of miscarriage [18, 19].

There is paucity of local literature with regards to incidence of depression after a miscarriage and the factors influencing its occurrence with no studies published with regards to its prevalence in the African population. Henceforth, we set out to determine the prevalence of depressive illness in patients after a miscarriage at a private tertiary teaching hospital in Kenya.

## Methods

### Objective

To determine the prevalence of positive depression screen for women who have experienced a miscarriage at the Aga Khan University Hospital Nairobi.

### Study design

The study was a cross-sectional study where we examined both the exposure to a miscarriage to the outcome of depression at the same time.

### Study setting and participants

The study was conducted at the Aga Khan University hospital Nairobi (AKUH, N). Participants were recruited from the Gynecology Clinics, which run daily, at the clinic review after a miscarriage. A miscarriage was defined as a pregnancy loss occurring at or before 23 weeks of a pregnancy or the miscarriage of a fetus of less than 500 g. Participants had some sort of treatment of the miscarriage including expectant, medical or surgical management. The clinic review was usually scheduled 2 weeks after the miscarriage but had a range of between one and 3 weeks. We further explore the choice of the study participants in the discussion.

### Inclusion and exclusion criteria

We included women in the reproductive age group (15–49 years) who had experienced a miscarriage which were either spontaneous or induced. We excluded other forms of early pregnancy losses like women with a diagnosed ectopic pregnancy in order to eliminate it compounding the analysis of the impact of the mode of treatment on prevalence of depression. Women with previously diagnosed depression were also excluded.

### Study procedures

Participants were required to sign consent to be included in the study after which two types of data were collected from the patient. A demographics tool and the Edinburgh Postnatal Depression scale (EPDS) [20] was administered to the patient. The data collection tools were administered by the principal investigator and a research assistant (a nurse in the gynecology clinic). Women were approached in the triage room after triage had been done. We approached a total of 202 women to get the sample size of 182 women – 20 women declined to be included.

### Study tools

The study used two tools to collect information from the patients – a demographics tool and the EPDS.

The demographics tool was created for the study which collected the patients file number, age and other associated factors that may impact on the occurrence of post-miscarriage depression. These factors included the patient’s age, educational background, gestation at miscarriage, marital status, planning of the pregnancy, social support, mode of treatment of the miscarriage, number of prior miscarriages, mode of conception and prior pregnancy outcome.

Depression symptoms were measured using the Edinburgh Postnatal Depression Scale (Additional file 1) [20]. This tool is a 10 item questionnaire that inquires on the presence of depressive symptoms in the period preceding the interview. Participant’s responses regarding the frequency of symptoms are scored from 0 to 3. Total score may range from 0 to 30 [17]. A score of 13 and above was deemed screen-positive and indicative of a high likelihood of depression as used in prior studies [17]. The checklist and the demographics tool were administered by the principal investigator or a research assistant to patients coming for the post miscarriage clinic review at the Gynecology clinic.

### Sample size and sampling method

There are no baseline prevalence studies from our population but from published literature from studies conducted worldwide but not in Africa [14] it is estimated that post miscarriage depression occurs in about 10–20% of women. More so, a study done in the AKUH, N using the EPDS for detection of postnatal depression in mothers revealed a prevalence rate of 13% [21]. This being the same setup we may assume the prevalence of post-miscarriage depression may likely be similar to the rate of postnatal depression and hence use it in the sample size calculation.

Sample size was calculated from a formula for estimating a population prevalence [22].

$$ \mathrm{n}=\frac{{\mathrm{Z}}^2\mathrm{P}\left(1\hbox{-} \mathrm{P}\right)}{{\mathrm{d}}^2} $$

Where: n = Required sample size. Z = Z statistic for a 95% confidence interval [1.96). P = Expected prevalence of Post miscarriage depression at AKUH, N. d = Precision around expected prevalence ±0.05. Substituting for the equation:

$$ {\displaystyle \begin{array}{l}\mathrm{n}=\frac{1{.96}^2\times 0.13\left(1\hbox{-} 0.13\right)}{0{.05}^2}\\ {}\mathrm{n}=173.\end{array}} $$

The patients were selected by a consecutive sampling method.

### Data management and analysis

The prevalence of positive depression screen for women who have experienced a miscarriage was determined as a percentage of patients who screened positive from the total participants. Descriptive statistics for continuous variables, such as age and gestation age at the miscarriage, were done to describe the baseline characteristics of women in the screen – positive and screen – negative groups. The statistical package for the social sciences (SPSS) version 22 was used for data analysis. Data were represented in tables and graphs.

### Ethical considerations

Ethical approval was obtained from the Research and Ethics committee at the AKUH, N. Participants were recruited after obtaining written informed consent as per study protocol. Patients had the right to refuse or withdraw from the study at any point and this did not impact on the quality of care received subsequently. The study used alternative study numbers to maintain patient privacy and confidentiality. The data collection forms were safely kept in a locked cabinet to which only the primary investigator and the research assistant had access to. Patient who screened positive for post-miscarriage depression had an appointment booked for further evaluation and management by the psychiatrist, this however was at their own cost. Around 80% (50 of the 62) of women referred attended a psychiatrist follow up, the number included the women who had suicidal ideation and they were offered appropriate counselling and follow up.

## Results

A total of 182 patients were recruited for the study. The patients were recruited from the outpatient gynecological clinics at the AKUH. No patients were excluded from this number since they all had their documentation in full.

### Patient characteristics

The age of the patients recruited varied from as low as 17 years to the oldest being 45 years old. The mean age was 29.42 (SD = 5.6) years as shown in Table 1.Most of the patients had a university/college level of education (87.4%; *n =* 159) with less having a primary level (2.2%; *n =* 4) or secondary level of education (10.4%; *n =* 19).

**Table 1:** Patient characteristics including age, gestational age at miscarriage, level of education, marital status, pregnancy planning, social support, others being aware of pregnancy, number of prior miscarriages, mode of conception and prior pregnancy outcome

Variable	Description	Value
Age	Mean (SD)	29.42 (±5.6) years
Gestational age at miscarriage in weeks	Mode (*n*)	8 weeks (41 women)
Level of education	Primary *n(*%)	4 (2.2%)
	Secondary *n(*%)	19 (10.4%)
	College/University *n(*%)	159 (87.4%)
Marital status	Single *n(*%)	34 (18.7%)
	Married *n(*%)	148 (81.3%)
Pregnancy planning	Planned *n(*%)	132 (72.5%)
	Unplanned *n(*%)	50 (27.5%)
Social Support	Lives alone *n(*%)	19 (10.4%)
	Lives with others *n(*%)	163 (89.6%)
Others aware of pregnancy	Yes *n(*%)	158 (86.8%)
	No *n(*%)	24 (13.2%)
Prior Miscarriage	None *n(*%)	124 (68.1%)
	1 *n(*%)	44 (24.2%)
	2 *n(*%)	6 (3.3%)
	3 *n(*%)	8 (4.4%)
Mode of Conception	Spontaneous *n(*%)	173 (95.0%)
	Assisted *n(*%)	9 (5.0%)
Prior Pregnancy Outcome	None *n(*%)	76 (41.8%)
	Miscarriage *n(*%)	38 (20.9%)
	Live Birth *n(*%)	68 (37.4%)

The gestational age at miscarriage varied from 3 weeks to the maximum of 23 weeks with the mode gestational age being 8 weeks(22.5%; *n =* 41). A large percentage (81.3%; *n =* 148) of the patients were married with also a high percentage of the pregnancies being planned (72.5%; *n =* 132). A high proportion of these women with miscarriages had social support with 89.6% (*n =* 163) living with other individuals and this corresponded to the high percentage of them having another individual aware of their pregnancy (86.8%; *n =* 158). This information is also displayed in Table 1. Furthermore, 68.1% (*n =* 124) of the women recruited had not experienced a previous miscarriage with about a quarter having experienced one prior miscarriage (24.2%; *n =* 44) and much less having had 2(3.3%; *n =* 6) or 3(4.4%; *n =* 8) prior miscarriages. Most women had conceived spontaneously (95%; *n =* 173) with 37.4% (*n =* 68) having had a live birth in their previous pregnancy (Table 1).

**Fig. 1:**
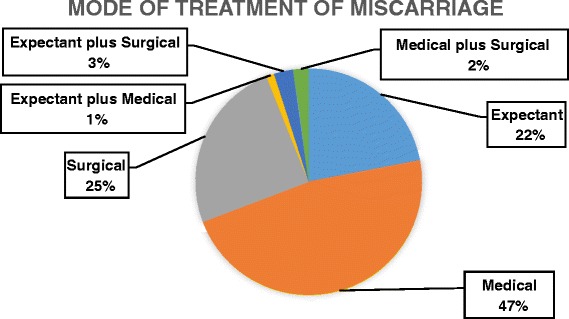
Mode of treatment of miscarriages

Almost half of the miscarriages seen were managed medically (47.3%; *n =* 86) with a small percentage (6%; *n =* 11) requiring a combination of more than one mode of *treatment* (Fig. 1).

### Prevalence of positive depression screen

**Fig. 2:**
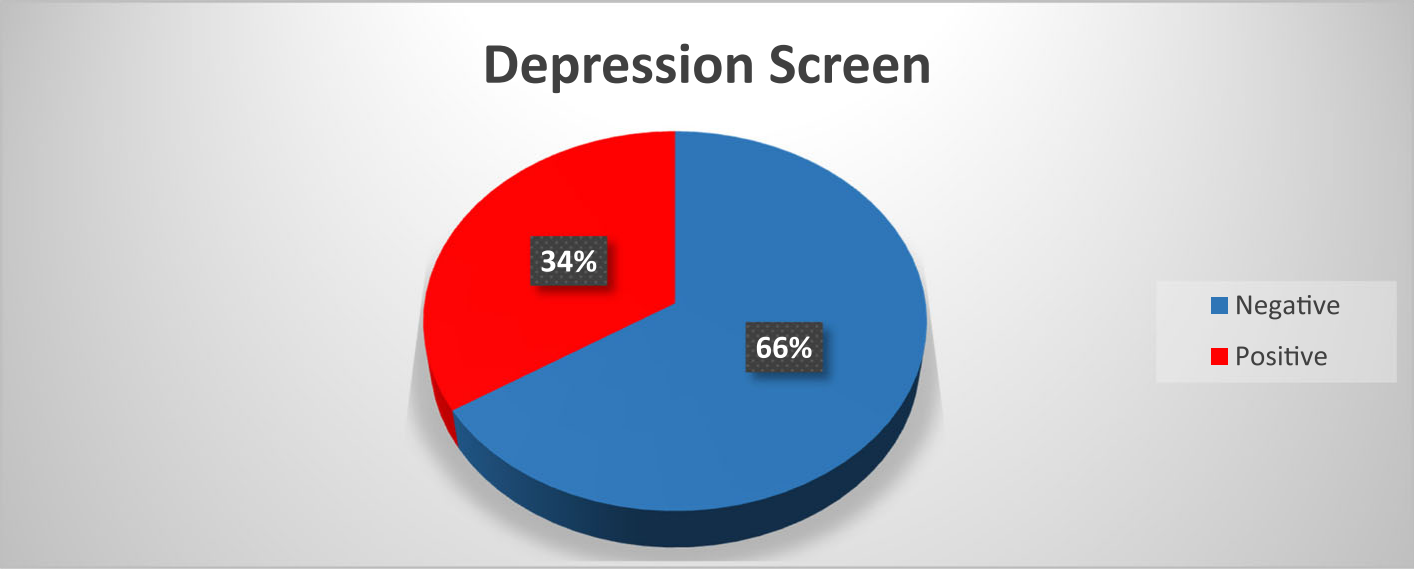
Depression screen result

The prevalence for positive depression screen in post miscarriage patients from the present study is estimated at 34.1%. In the recruited patients, 62 of the 182 participants scored 13 or more in the EPDS and had a positive screen for post miscarriage depression (Fig. 2). Patients who had a positive depression screen were referred for psychiatric review at their convenience and cost. This study did not envisage further follow up of these participants.

### Self-harm

**Fig. 3:**
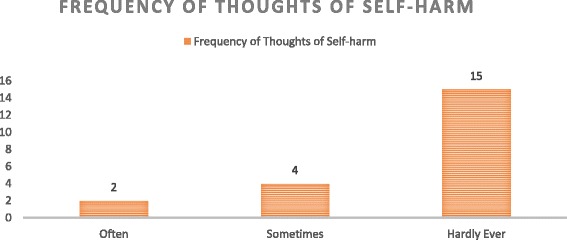
Frequency of Thoughts of Self harm

The last question of the EPDS assesses thoughts of self-harm and because of it implication it’s important to highlight the outcome result of this question. Out of the 62 participants who had a positive depression screen, 21(33.8%) had thoughts of self-harm which were distributed as shown in Fig. 3.

## Discussion

Miscarriages are common especially in early pregnancy and with the growing concern about its mental health implications, it’s an area of growing interest in obstetrics and gynaecology. The present study set out to determine the prevalence of positive depression screen among women who have had a miscarriage.

The present study recruited 182 women who had miscarriage and screened them after having had treatment for the miscarriage. The patients recruited had a mean age of 29.42 years with the youngest being 17 years and the oldest 45 years. More so, although it is known that miscarriages tend to increase with advancing age [23], most of our patients were mainly 26 to 31 year olds which represented 47.3% of all the patients. This can be attributed to the particular demographic the hospital serves which includes young working women and hence may not be a true representation of the occurrence of miscarriages with age.

The mode gestational age at miscarriage was 8 weeks. This is in keeping with previous studies in miscarriages as most are seen within the first trimester and tend to reduce with advancing age [24]. This pattern also been documented in a local study that documented miscarriages with advancing gestational age [4]. It was observed that their peak age of miscarriages was at 6 weeks with around 25% of the pregnancies observed resulting in miscarriage. The cause of the high incidence of miscarriage in early pregnancy has not well elucidated although there is increasing concern that it may be as a result of disorders in genetic makeup of the pregnancy and placentation with low levels of angiogenic markers also playing a key role [25].

Most of women recruited in this study had an advanced level of education with 87.4% having a college or university level education. This is in keeping with studies done in pregnant women from a similar population. Majority of the women recruited were married (81.3%) and more of their pregnancies were planned (72.5%). This again just points out to the social strata of most patients with a miscarriage in our setting [4]. In the present study, 89.6% of the women recruited had social support meaning they did not live alone and consequently, 86.8% reported that the pregnancy ending in miscarriage was known to another person. This again points out to the social setting of the women recruited with expectations that most of them would plan a pregnancy after marriage and hence wouldn’t be living alone and probably a another person would know about their miscarriage. This in turn may impact on the occurrence of prevalence found in this population, we may postulate that this prevalence may be different in a population with a lesser proportion of social support.

The prevalence of positive depression screen in post miscarriage women in our study was 34.1%. Sixty two of the 182 women had an EPDS score of 13 or more. This is the highest prevalence of positive depression screen after a miscarriage reported in recent literature. Previous studies reported a prevalence ranging from as low as 10% to as high as 28.7% [14, 15]. The high prevalence in our study is a novel finding with regards to depression after a miscarriage. The occurrence of depression after a miscarriage has been strongly attributed to socio-cultural beliefs [26, 27]. More so, in the African context many myths exist in regards to the cause and future implications of miscarriage [16]. These myths do influence the ability of a woman to seek psychological help after a miscarriage and go through the proper grief process and subsequently explain the high prevalence of depression after a miscarriage [26]. Compared to more developed western societies, where a woman experiencing a miscarriage may understand better its cause and implications to future pregnancies [23], the weight of African socio-cultural beliefs may impact on the psychological morbidity experienced after a miscarriage hence the high prevalence observed. The interactions of the socio-demographic factors and the occurrence of depression after a miscarriage are discussed in a subsequent manuscript that’s a follow up to the current one.

Thoughts of self-harm were observed in about a third (33.8%; *n =* 21) of the women who screened positive for post miscarriage depression. Although it was noted that most of the women hardly ever had these thoughts, it is concerning that they are present in the setting of post miscarriage depression. No studies have been done in post miscarriage women assessing their thoughts of self-harm, however in postnatal women assessed with the EPDS the prevalence reported varies from around 4–20% [28, 29]. The occurrence of self-harm was further correlated to risk factors such as younger maternal age, being single, history of childhood abuse and associated symptoms of sleep disturbance and anxiety [28, 29]. Although the present study was not powered to investigate these factors, we can posit that the same factors may play a role in women with a positive depression screen after a miscarriage.

### Limitations and future directions

The limitations of the study included not being able to confirm clinical depression using a psychiatric consult which was due to financial constraints due to a limited budget. But this may be a study to undertake now that we have shown that post miscarriage depression is an area of concern in the field of obstetrics and gynaecology. The other limitation was lack of further follow up of the women who had a positive depression screen to elucidate whether this number waned off with passing time. This would have aided in further understanding of the psychological evolution of the depressive episode. This was also due to the constraint of time the study was to be carried out in but we hope further studies can be done to elucidate this in our population.

## Conclusion

In conclusion, a positive depression screen is present in 34.1% of women in our population 2 weeks after a miscarriage. More so, factors that seem to impact on the positive depression screen include a younger age, a lower education level, an older gestational age at miscarriage, being single, an assisted mode of conception and a prior miscarriage – these have been further explored in a subsequent manuscript. Thoughts of self-harm are present in about a third of these women (33.1%) hence pointing out the importance of screening these women using the EPDS after a miscarriage. The prevalence documented in this study outlines the importance of screening of women after a miscarriage for depression, more so, with associated self-harm in a third of these women this further strengthens the argument for universal screening. With universal screening, earlier pick up of these women may reduce the subsequent well documented effects of depression and primarily offer better universal care after a miscarriage. Moreover, the role of universal counselling after a miscarriage and its impact on reduction of depression needs to be explored in the clinical setting.

## Additional file

Additional file 1: The Edinburgh postnatal depression scale. The EPDS is the tool used in the present study to screen for post-miscarriage depression. (DOCX 14 kb)
