# Supplementary material for: Prevalence of positive depression screen among post miscarriage women- A cross sectional study
Source: BMC Psychiatry. 2018 Feb 5;18:32. doi: 10.1186/s12888-018-1619-9 (PMC5799918; doi:10.1186/s12888-018-1619-9)
Supplement: Additional file 1: — The Edinburgh postnatal depression scale. The EPDS is the tool used in the present study to screen for post-miscarriage depression. (DOCX 14 kb) [file 12888_2018_1619_MOESM1_ESM.docx]

## Appendix 4: The Edinburg Postnatal Depression Scale

As you have recently had a miscarriage, we would like to know how you are feeling now. Please underline the answer which comes closest to how you have felt in the past seven days, not just how you feel today.

Here is an example, already completed:

I have felt happy:

[ ]Yes, most of the time

[Χ]Yes, some of the time

[ ]No, not very often

[ ]No, not at all

This would mean: “I have felt happy some of the time” since the miscarriage.

Please complete the other questions in the same way.

**SINCE THE MISCARRIAGE**

1. I have been able to laugh and see the funny side of things:

[ ] As much as I always could

[ ] Not quite so much now

[ ] Definitely not so much now

[ ] Not at all

1. I have looked forward to enjoyment in things:

[ ] As much as I ever did

[ ] Rather less than I used to

[ ] Definitely less than I used to

[ ] Hardly at all

1. I have blamed myself unnecessarily when things went wrong:

[ ] Yes, most of the time

[ ] Yes, some of the time

[ ] Not very often

[ ] No, never

1. I have felt worried and anxious for no very good reason:

[ ] No, not at all

[ ] Hardly ever

[ ] Yes, sometimes

[ ] Yes, very often

1. I have felt scared or panicky for no very good reason:

[ ] Yes, quite a lot

[ ] Yes, sometimes

[ ] No, not much

[ ] No, not at all

1. Things have been getting on top of me:

[ ] Yes, most of the time I haven‟t been able to cope at all

[ ] Yes, sometimes I haven‟t been coping as well as usual

[ ] No, most of the time I have coped quite well

[ ] No, I have been coping as well as ever

1. I have been so unhappy that I have had difficulty sleeping:

[ ] Yes, most of the time

[ ] Yes, sometimes

[ ] Not very often

[ ] No, not at all

1. I have felt sad or miserable:

[ ] Yes, most of the time

[ ] Yes, quite often

[ ] Not very often

[ ] No, not at all

1. I have been so unhappy I have been crying:

[ ] Yes, most of the time

[ ] Yes, quite often

[ ] Only occasionally

[ ] No, never

1. The thought of harming myself has occurred to me:

[ ] Yes, quite often

[ ] Sometimes

[ ] Hardly ever

[ ] Never

Reviewer ……………………………………….Total score ………. Date ………………………

Source: (24) Cox JL, Holden JM, Sagovsky R. Detection of postpartum depression. Development of the 10-item EPDS. Br J Psychiatry. 1987 Jun; 150:782-6.
